# Graded multilayer triple cation perovskites for high speed and detectivity self-powered photodetector via scalable spray coating process

**DOI:** 10.1038/s41598-022-14774-x

**Published:** 2022-06-30

**Authors:** Koth Amratisha, Waris Tuchinda, Pipat Ruankham, Atittaya Naikaew, Pimsuda Pansa-Ngat, Ladda Srathongsian, Worawat Wattanathana, Ko Ko Shin Thant, Ratchadaporn Supruangnet, Hideki Nakajima, Somboon Sahasithiwat, Pongsakorn Kanjanaboos

**Affiliations:** 1 School of Materials Science and Innovation, Faculty of Science, Mahidol University, Nakhon Pathom, 73170 Thailand; 2 Department of Physics and Materials Science, Faculty of Science, Chiang Mai University, Chiang Mai, 50200 Thailand; 3 Research Center in Physics and Astronomy, Faculty of Science, Chiang Mai University, Chiang Mai, 50200 Thailand; 4 Department of Materials Engineering, Faculty of Engineering, Kasetsart University, Bangkok, 10900 Thailand; 5 Synchrotron Light Research Institute (Public Organization), Nakhon Ratchasima, 30000 Thailand; 6 National Metal and Materials Technology Center (MTEC), National Science and Technology Development Agency, Pathum Thani, 12120 Thailand; 7 Center of Excellence for Innovation in Chemistry (PERCH-CIC), Ministry of Higher Education, Science, Research and Innovation, Bangkok, 10400 Thailand

**Keywords:** Sensors and biosensors, Optical materials and structures

## Abstract

Rapid advancements in perovskite materials have led to potential applications in various optoelectronic devices, such as solar cells, light-emitting diodes, and photodetectors. Due to good photoelectric properties, perovskite enables low-cost and comparable performance in terms of responsivity, detectivity, and speed to those of the silicon counterpart. In this work, we utilized triple cation perovskite, well known for its high performance, stability, and wide absorption range, which is crucial for broadband photodetector applications. To achieve improved detectivity and faster response time, graded multilayer perovskite absorbers were our focus. Sequential spray deposition, which allows stacked perovskite architecture without disturbing lower perovskite layers, was used to generate single, double, and triple-layer perovskite photodetectors with proper energy band alignment. In this work, we achieved a record on self-powered perovskite photodetector fabricated from a scalable spray process in terms of EQE and responsivity of 65.30% and 0.30 A W^-1^. The multilayer devices showed faster response speed than those of single-layer perovskite photodetectors with the champion device reaching 70 µs and 88 µs for rising and falling times. The graded band structure and the internal electric field generated from perovskite heterojunction also increase specific detectivity about one magnitude higher in comparison to the single-layer with the champion device achieving 6.82 × 10^12^ cmHz^1*/*2^ W^*−*1^.

## Introduction

With the increasing popularity of perovskite solar cell technology, there have been many attempts to utilize perovskite’s good photoelectric properties, such as high absorption coefficient, carrier mobility, bandgap tunability, and most importantly, low-cost production to fabricate many types of photoelectronic devices, apart from solar cells^1–6^. Perovskite materials can be processed with solution-based, low-cost fabrication methods i.e. spin coating^7–11^, spray coating^12–14^, dip coating^15^, and slot die coating^16^. One of the most interesting applications for perovskite-based devices is electromagnetic wave detection. In terms of responsivity, detectivity, and speed, perovskite photodetector (PPD) has exhibited comparable or even better performances, relative to its silicon or InGaAs counterparts^17–19^. Photodetector has many distinct architectures, but the most common ones are p-n, p-i-n, and m-s-m junction-driven photodiodes^20–22^. For the inorganic-based photodetectors to achieve high external quantum efficiency (EQE) and responsivity, external bias is required to drive electron–hole pair apart, thus promoting charge extraction and preventing charge recombination^23–25^. However, the external bias increases the operation cost of silicon PDs, rendering them unsuitable in some applications such as low-power integrated devices for the internet of things. To improve upon the self-powered performances of silicon-based PD, the Schottky effect from heterojunction between silicon and graphene was exploited^20,26^. Similarly, photovoltaic performance of self-powered PPDs can be enhanced with the integration of electron and hole transporting layers (ETL and HTL). Conventionally, ETLs and HTLs have been fabricated with a simple solution-processable, which keeps the fabrication costs of PPDs relatively low^23,25,27^. While both silicon and perovskite photodetectors are operable without external bias, silicon detectors are known for outputting high power density while the perovskite counterpart generally has a significant edge in detector speed and fabrication cost^17–19^.

Perovskite molecule structures are oftentimes denoted as ABX_3_, where A = Cs^+^, CH_3_NH_3_^+^ (MA^+^), HC(NH_2_)_2_^+^ (FA^+^), B = Pb^2+^, Sn^2+^, Ge^2+^, and X = I^−^, Br^−^, Cl^-^. MAPbI_3_ is the most common formula for perovskite solar cells. Without the help of ETL and HTL, self-powered MAPbI_3_–based PPDs have been reported to detect electromagnetic waves in the range between 400 to 900 nm, with rise and fall times of 30 μs and 300 μs, respectively. The reported device exhibited the responsivity of 0.26 A W^-1^^28^. A strategy to improve upon the performance of self-powered PPDs, without the integration of ETL and HTL, is the utilization of multiple perovskite layers to create graded band structures. Properly graded energy band levels can result in the formation of internal built-in potentials, which promotes charge carrier extraction.

Aligning semiconducting materials with different work functions results in the formation of a depletion zone at the interface due to charge migration in an attempt to keep the Fermi level equal across the materials^29^. The depletion zone houses the electric field caused by energy band shifting, which improves charge transfer through a device^21,30–32^. Furthermore, the internal potential from the junction could reduce the dark current of photodetector devices, which equates to a reduction in signal noise and an increment in the on/off signal ratio—higher detectivity^33^. Research by Cao et al.^34^ showed that MAPbBr_3_ and MAPbI_x_Br_3−x_ heterojunction could be created by dipping a MAPbBr_3_ single crystal in a MAPbI_3_ precursor solution. MAPbBr_3_/MAPbI_x_Br_3−x_ heterojunction can improve EQE and responsivity from 0.14% to 3.17% and 0.0095 A W^-1^ to 0.0115 A W^-1^, respectively. N-type MAPbI_3_ could also be doped on the surface with MoO_3_, as demonstrated by Ou et al.^30^, creating homojunction between pristine MAPbI_3_ and p-type MoO_3_-doped MAPbI_3_ at the surface. When compared to the non-graded counterpart, the homojunction structure exhibited improvements in EQE from 0.20% to 3.93% and responsivity from 1.42 A W^-1^ to 3.93 A W^-1^. While PPDs with multiple perovskite layers are superior to their single-layer counterparts, it is undeniable that the junction between perovskite and ETL still greatly influences the performances of PPDs. As shown by Li et al.^35^, the ZnO/CsPbBr_3_ PPD achieved 11.5 mA W^-1^, which is significantly higher than those of CsPbBr_3_ PPD without the ZnO ETL, which exhibited only 0.10 mA W^-1^ in responsivity. Therefore, integration of both transporting layers and graded band structures should be done. Aside from direct photovoltaic performance, perovskite lacks long-term stability under oxygen, humidity, heat, UV, and light illumination, due to their low formation energy^8,36–38^. To date, triple cation materials such as Cs_0.05_FA_0.79_MA_0.16_PbI_2.52_Br_0.48_ are among the best perovskite recipes in terms of both photoelectric performance and stability. Recent progress saw triple cation perovskite replacing the MAPbI_3_ formula^39–41^. In previous research, triple cation self-powered photodetectors with ETL and HTL were fabricated through spin coating, achieving rise and fall times of 19 μs and 21 μs, respectively, and responsivity of 0.52 A W^-1^ at 1 sun covering 350 nm to 800 nm wavelength of light^23^. The method of creating heterojunction or homojunction via doping at the surface is popular for improving performance in the photovoltaic device^21^. However, the heterojunction between different types of perovskite was difficult to fabricate. The precursor solution from the upper layer can dissolve the lower perovskite already deposited. In the previous work, sequential spray deposition (SSD) was developed to fabricate multilayer perovskite solar cells where a precursor solution from an upper-layer perovskite deposition did not dissolve a lower layer perovskite^42^. Additionally, in the case of real-world production, the spray process is more promising due to scalability and larger coating area per precursor solution used. As a strategy to obtain different perovskite materials for graded band structures, different ratios of Cs cations in the triple cation perovskite structure were used, effectively changing work functions and band energies^41,43^. In this work, Cs_0.05_FA_0.79_MA_0.16_PbI_2.52_Br_0.48_ (5%Cs), Cs_0.1_FA_0.75_MA_0.15_PbI_2.52_Br_0.48_ (10%Cs), and Cs_0.15_FA_0.71_MA_0.14_PbI_2.52_Br_0.48_ (15%Cs) with distinct band positions were used as LEGO brick components to construct stacked PPD architecture. As Cs^+^ content increases, the valence band maximum becomes closer to the Fermi level, making our stacking layers gradually more p-type towards HTL^41,44^.

## Methodology

Lead(II) iodide (PbI_2_; 99% purity), lead(II) bromide (PbBr_2_; 98% purity), formamidinium iodide (FAI; ≥ 99% purity, anhydrous), methylammonium bromide (MABr; 99.99% purity), cesium iodide (CsI; 99.9% trace metals basis), anhydrous N,N-dimethylformamide (DMF; 99.8% v/v), anhydrous dimethyl sulfoxide (DMSO; 99% v/v), anhydrous ethanol (99.5% v/v), tin(II) chloride dihydrate (SnCl_2_·2H_2_O; 99.999% purity), and hydrochloric acid (HCl; 37% v/v) were purchased from sigma-aldrich.

SnCl_2_·2H_2_O solution (0.3 M) was prepared in anhydrous ethanol under ambient conditions and stirred at room temperature for at least 1 h before filtering with a PTFE syringe filter (Whatman, 0.22 mm). Cleaned ITO substrates were deposited with the precursor solution using spin deposition with a spin speed of 2000 rpm and 1000 rpm s^-1^ acceleration. The deposited films were then placed on a 70 °C hotplate before heating up to 180 °C and held for 1 h.

Triple cation perovskite Cs_0.05_FA_0.79_MA_0.16_PbI_2.52_Br_0.48_ (5%Cs), Cs_0.1_FA_0.75_MA_0.15_PbI_2.52_Br_0.48_ (10%Cs), and Cs_0.15_FA_0.71_MA_0.14_PbI_2.52_Br_0.48_ (15%Cs) precursor solutions were prepared from weighing the different chemical powders according to the stoichiometric ratio. The mass of each powder for 1 mL solution are as follows: 5%Cs: CsI 15.6 mg, MABr 21.5 mg, FAI 163.0 mg, PbBr_2_ 70.5 mg, PbI_2_ 478.5 mg; 10%Cs: CsI 31.2 mg, MABr 20.2 mg, FAI 154.8 mg, PbBr_2_ 72.7 mg, PbI_2_ 461.9 mg; 15%Cs: CsI 46.8 mg, MABr 18.8 mg, FAI 146.5 mg, PbBr_2_ 74.9 mg, PbI_2_ 459.2 mg. Note that additional 2.5% of excess cation salt were added to mitigate PbI_2_ phase in perovskite film. The powders were then dissolved in DMF/DMSO mixed solvent with ratio of 4:1, respectively under N_2_ glovebox environment. The solutions were stired using a hotplate stirrer at room temperature for at least 3 h before filtering with PTFE syringe filter.

Airbrushes (badger 200 series connected to N_2_ gas with 30 psi) under ambient environment (40–50%RH) were utilized. For all spray depositions, substrates were pre-heated to 110 °C before and during spray deposition. For single-layer perovskite deposition, we used a single airbrush with a spray rate of 50 μL s^-1^; the airbrush moved at 12 mm s^-1^ while depositing the precursor solution on the substrate for 3 times. For double-layer perovskite, the airbrush speed was 8 mm s^-1^, 1 time for each layer. For triple-layer perovskite, the airbrush speed was 12 mm s^-1^, 1 time for each layer. The films were left on a hot plate for 30 s between layers.

The spiro-OMeTAD solution was prepared by mixing 80 mg of spiro-OMeTAD in 1 mL of chlorobenzene at room temperature for 1 h. After that 28.5 μL of 4-*tert*-butylpyridine and 17.5 μL of Li-TFSI solution (520 mg/mL in acetonitrile) were added and stirred overnight. The solution was dropped on a perovskite film and rested for 30 s before spin coating with a spin speed of 2,000 rpm and acceleration of 1,000 rpm s^-1^. Finally, the film was removed from the glovebox for spiro-OMeTAD oxidation for 2–5 min before putting back in the glovebox.

The optical absorption spectra were recorded using a Shimadzu UV-2600 UV–Vis spectrophotometer (900–300 nm, slow mode, and absorbance mode). The crystal structure was characterized by a DKSH Aeris benchtop X-ray diffractometer (Cu anode material, detector scan mode using a step size of 0.0217°, 0.98 s per step, and 2θ starts from 5° to 50°). The perovskite film stability was studied using Bruker, D8 Discover X-ray diffractometer (Cu anode material, detector scan mode using a step size of 0.02°, 198.7337 ms per step, and 2θ from 5° to 50°). Surface morphologies and cross-sections were observed by scanning electron microscopy (SEM; Quanta 450 FEI, tungsten filament electron source, 20 kV, and secondary electron mode). Elemental EDS mapping of cross-sectional images were analyzed by field emission scanning electron microscope JSM-7610FPlus, JEOL FE-SEM with EDS detector ULTIM MAX 65 from Oxford Instruments. Grazing incidence X-ray diffraction (GIXRD) was performed in a PANalytical Empyrean diffractometer. For the wide scan, the grazing angle (*ω*) was scanned from 0.2° to 1.2°, 2θ measurement range of 10°–45°. The scan step was set to 0.02°/step and the measurement time per step was 147.39 s. For the narrow scan, the grazing angle (*ω*) was scanned from 0.2° to 0.8° in 2θ measurement range of 13°–15°, scan step was set to 0.02°/step, and the measurement time per step was 10.56 s. Ultraviolet photoelectron spectroscopy was measured at Synchrotron Light Research Institute (public organization), Thailand. Photoluminescence of samples was measured by Horiba FluoroMax Plus spectrofluorometer (integration time of 0.1 s, excitation of 480 nm, excitation slit of 15 nm, emission wavelength measurement between 650 and 900 nm, and emission slit of 10 nm). Photoluminescence lifetime (PL-lifetime) of the samples was measured by Horiba Jobin Yvon FluoroMax 4 spectrofluorometer (time-correlated single photon counting unit). A nanoLED diode emitting pulses at 625 nm was used as an excitation source with band pass of 8 and sync delay of 60 ns. For data acquisition, the measurement range was 100 ns with coaxial delay of 90 ns. EQE, responsivity and specific detectivity were measured using Enlitech QE-R quantum efficiency analyzer (DC mode with 1 mm^2^ beam diameter). For photoresponse measurements, the PPDs were exposed to 1 sun illumination with an optical chopper spinning at 1000 Hz while measuring the photocurrent with Tektronix TBS1072b-EDU oscilloscope. Atomic force microscope (Park NX10 AFM) was used to measure the surface morphology of perovskite film. The z height and the modulus mapping of the films were constructed using the pinpoint mode.

## Results and discussion

**Figure 1:**
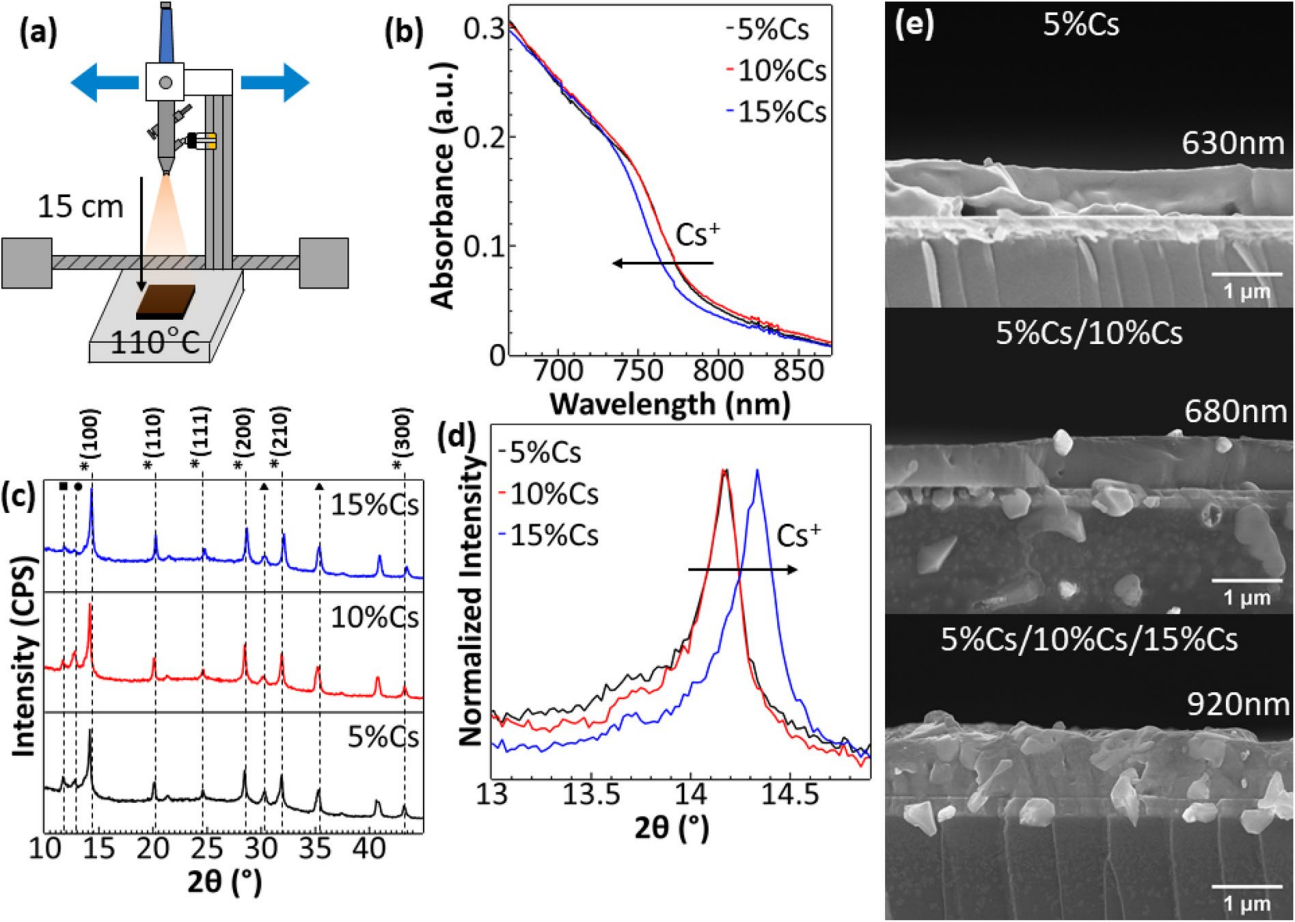
(**a**) Schematic of spray deposition setup used in this research. The airbrush was attached to the screw which was driven by a stepping motor, which moves the airbrush left and right on top of the hot plate. (**b**) UV–Visible spectra at the absorption edge of triple cation perovskite films with different Cs^+^ content. (**c**) X-ray diffraction spectra (XRD) of triple cation perovskite (labeled with *) films with different Cs^+^ contents. PbI_2_ peaks were labeled with •. Optically inactive FAPbI_3_ peaks were labeled with ▪. ITO substrate peaks were labeled with ▴^39,45,46^. (**d**) XRD spectra of triple cation perovskite films at the 2θ from 13° to 15°. (**e**) SEM cross-sections of single (5%Cs), double (5%Cs/10%Cs), and triple-layer (5%Cs/10%Cs/15%Cs) perovskite films.

Triple cation perovskite films were deposited on ITO/SnO_2_ substrate using the spray setup shown in Fig. 1a. We improved the spray methods from the previous work^42^ by attaching an airbrush to a moving rail. During spray deposition, the airbrush’s constant movement provides a constant flux of precursor droplets, creating a more homogenous surface finish along the direction of spray movement and covering larger active areas for PPDs. The single-layer perovskite was done by depositing three layers of one type of perovskite with an airbrush moving at the speed of 12 mm s^-1^ with 30 s of drying between layers. For the double-layer, the airbrush speed was reduced to 8 mm s^-1^ to compensate for fewer layers of perovskite. Two layers of different perovskite materials were deposited with the corresponding precursor solutions to create a graded structure. For the triple-layer, the airbrush speed was set to 12 mm s^-1^ and each layer was deposited with different precursor solutions. The 12 mm s^-1^ airbrush speed was chosen because this speed gave the best performance in terms of photoresponse as shown in Fig. S1 (Supporting material). When spraying, the substrate turned dark immediately after contact with spray droplets, indicating that the precursor solution evaporated very fast and had no time to dissolve the lower layer. The perovskite film from the spray process has a matt finish, indicating a rough surface. By observing the surface with an AFM, the coffee mark from each droplet can be seen as shown in Fig. S2; the surface modulus are shown in Fig. S3. Note that the spray deposition methods are very simple and can be easily adjusted for the larger substrates by changing the movement of the airbrush, which is suitable for use in a large-scale production.

The increase in Cs^+^ caused a blue-shift of the absorption edge, as shown in Fig. 1b which was the result of the slight shift in bandgap from 1.60 eV of 5%Cs and 10%Cs perovskite to 1.61 eV of 15%Cs perovskite. In Figure 1c, X-ray diffraction spectra show good crystallinity with correct peak positions at 14.18°, 20.12°, 24.59°, 28.48°, 31.89°, and 43.22° which correspond to (100), (110), (111), (200), (210), and (300) planes of the perovskite films, respectively^23,45^. The increase of Cs^+^ content in the crystal structure shifts the diffraction peaks of the perovskite corresponded peaks slightly to the higher angles, for example, the 14.18° peak in Fig. 1d. The slight shift in the diffraction peak was the result of a reduction in lattice parameter as smaller Cs^+^ replaced larger MA^+^^47^. Moreover, the small peak at 11.7°, which belongs to optically inactive FAPbI_3_, becomes smaller as the Cs^+^ content increases. The reduction of FAPbI_3_ with higher Cs^+^ content is the result of smaller Cs/FA/MA cation effective radius, which shifts the tolerant factor toward cubic lattice rather than the hexagonal phase of FAPbI_3_^39,48^. A very small peak at 12.79° corresponds to PbI_2_ crystal, pointing to incomplete formation of perovskite. The additional 2.5% of cation salt by weight is necessary to reduce the PbI_2_ in perovskite crystal. Without the additional cation, PbI_2_ in perovskite crystal was more significant. However, too much additional cation can also affect the performance. As shown in Fig. S4, the increase of cation reduces the performance of PPDs with 2.5% cation being the best performance with the lowest PbI_2_ peak.

To determine the effectiveness of SSD for depositing multilayer perovskite films without layer dissolution, cross-section images were captured as shown in Fig. 1e. The dissolution of the perovskite layer can occur both when depositing the same layer for multiple times and when depositing a new layer on top of the finished layer. During spray deposition, the precursor solution should evaporate as soon as the precursor solution hit the heated substrate. If the rate at which the solution is sprayed on the substrate is greater than the evaporation rate of the solvent, then the precursor solution can build up and dissolve the already crystallized film below, which we should refer to as “same layer dissolution”. A similar thing can occur on the deposited lower layer while spraying the upper layer. The precursor solution of the upper layer with too much spray rate can dissolve the lower film which we should refer to as “lower layer dissolution”. The lower layer dissolution is much more problematic than same layer dissolution as the interface between two types of perovskites can be difficult to determine if the materials are dissolved, mixed, and recrystallized into a composite. Moreover, the interface between perovskite layers may have unwanted defects which can reduce the performance of the PPD. The first cross-section was from 5%Cs with 4 mm s^-1^ spray speed for 1 time. The second cross-section was 5%Cs/10%Cs with 8 mm s^-1^ for each layer. The last cross-section was triple-layer 5%Cs/10%Cs/15%Cs with 12 mm s^-1^ for each layer. These spray speeds were calculated such that equal total spray time was performed for each condition, resulting in similar solutions being bombarded on the substrate. Any dissolution from deposition of the upper layer would cause a reduction of thickness when compared to that of a single-layer perovskite with the same total spray time. The thickness of the double-layer is thicker than that of the single-layer. Similarly, triple-layer perovskite is thicker than double-layer and single-layer ones, confirming little to no dissolution from depositing the top layer. The significant increase in thickness of the triple-layer perovskite film was the result of less “lower layer dissolution” and slightly naturally thicker 15%Cs perovskite film as shown in Fig. S5. The thicker double and triple-layer perovskite films confirmed the idea of using SSD as an effective deposition method to fabricate double and triple-layer perovskite films with an acceptable interface between perovskite layers.

**Figure 2:**
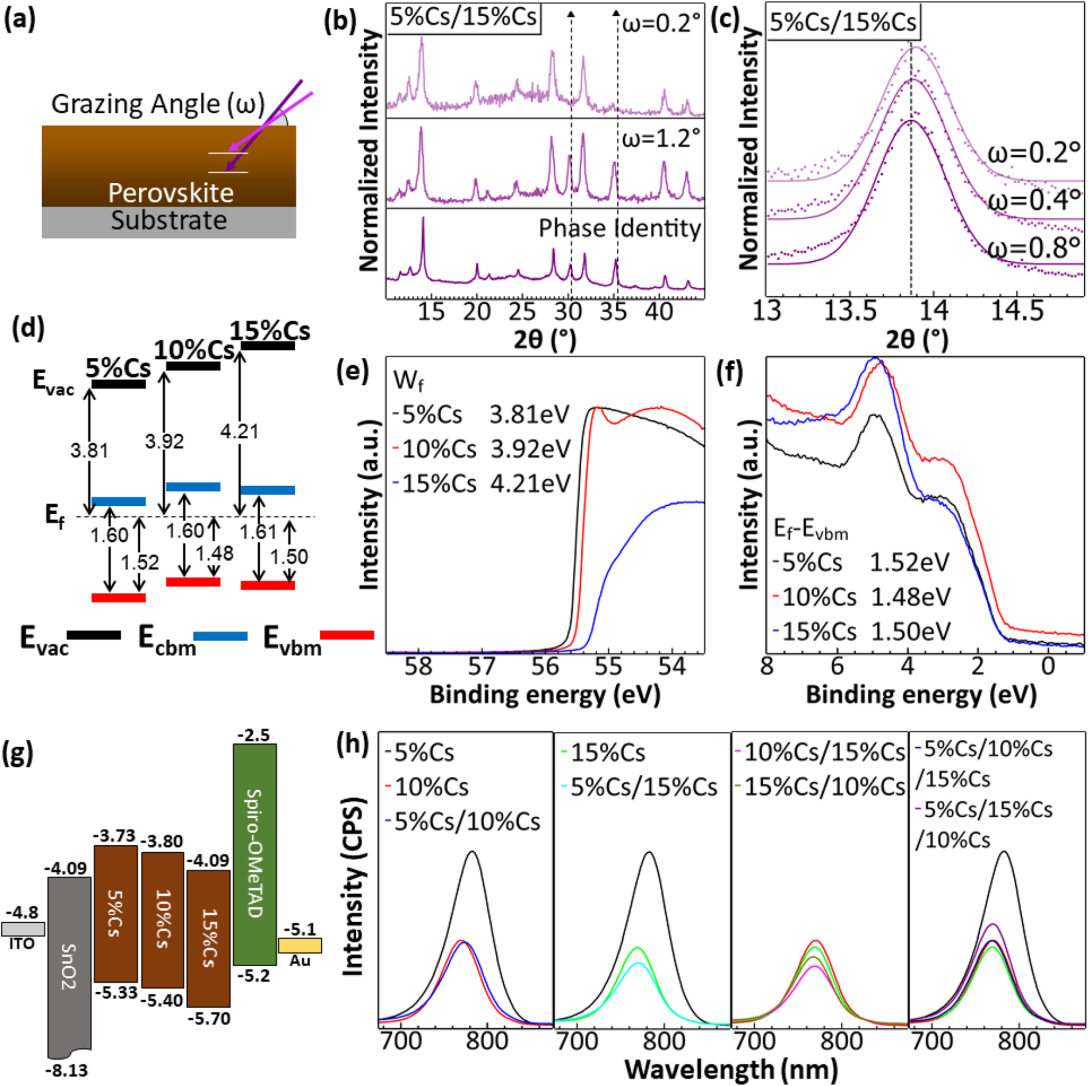
(**a**) Grazing incident X-ray diffraction (GIXRD) scanned at a deeper level with a larger grazing angle (ω) (**b**) GIXRD spectra of 5%Cs/15%Cs with different grazing angles. Phase identity is equivalent to that from the normal XRD. (**c**) GIXRD spectra of 5%Cs/15%Cs at the 2θ from 13° to 15° with guassian fits to identify X-ray diffraction peaks. (**d**) Band diagrams of 5%Cs, 10%Cs, and 15%Cs perovskites aligned by having E_f_ equals to zero, representing band alignment when perovskite films come in contact with each other. (**e**) Ultraviolet photoelectron spectroscopy (UPS) spectra at the secondary electron cutoff region and (**f**) at the valence band region of 5%Cs, 10%Cs, and 15%Cs. (**g**) Band diagrams of 5%Cs, 10%Cs, and 15%Cs aligned by having E_vac_ equals to zero^45,49^. (**h**) Photoluminescence (PL) spectra of single, double, and triple-layer perovskite films showing quenching of multi-layer perovskite films compared to that of single-layer perovskite films.

Grazing incident X-ray diffraction spectroscopy (GIXRD) can be used to observe material compositions at different depths. In Fig. 2a, the depth of the observed materials is dependent on a grazing angle. The GIXRD spectra in Fig. 2b show that at the 0.2° grazing angle no peak from ITO substrate was observed; at the low angle, the X-ray only scanned the top surface of the perovskite film. However, at the grazing angle of 1.2°, we observed X-ray diffraction peaks from the ITO substrate at 2θ around 30.5° and 35.5°, indicating deeper scanning depth. In Fig. 2c, the gaussian fits of the GIXRD spectra were used to determine the peak positions of a double-layer 5%Cs/15%Cs perovskite film with grazing angles of 0.2°, 0.4°, and 0.8°. The positions of 2θ of the perovskite peak near 14° were reducing as the grazing angles increased. As shown previously in Fig. 1d, the higher Cs^+^ content in triple cation perovskite resulted in larger 2θ at around 14°, indicating more Cs^+^ content at the top than at the bottom, confirming the expected grading structure of the multi-layer perovskite film. It is worth noting that the GIXRD used in Fig. 2b, c was acquired by a different machine from the one used in Fig. 1c, d, so the perovskite peaks were slightly shifted. We also made an elemental analysis at the cross-section of the double-layer perovskite film. However, the spatial error was too large to resolve shifted compositions as shown in Fig. S6.

Figure 2d shows the band diagram from UPS data shown in Fig. 2e, f. The UPS measurements were done under high vacuum condition, which may not accurately represent real-world condition under ambient. However, we can use the result to understand the performance of PPDs. E_f_-E_vbm_ determines valence band maximum (E_vbm_) relative to the Fermi level and the majority carrier of the material^50^. When semiconductors make electrical contact, the Fermi levels align and take the same value across the device. However, E_f_–E_vbm_ remains constant, altering E_vbm_ position as shown in Fig. 2d. E_vbm_ of 5%Cs, 10%Cs, and 15%Cs perovskites were 1.52 eV, 1.48 eV, and 1.50 eV, respectively. We observed an increase of E_f_-E_vbm_ for 15%Cs. When more Cs^+^ are incorporated in the materials, E_f_-E_vbm_ increases until some point which E_f_-E_vbm_ decreases as Cs^+^ continues to increase, agreeing with the previous publication^41^. The contact between 5 and 10%Cs creates a depletion zone which improves charge transfer and potentially increases the speed of the photodetector as well. The E_cbm_ and E_vbm_ of 10%Cs were higher than those of 5%Cs, creating a type II band alignment, which promotes electron moving from higher E_cbm_ of 10%Cs to lower E_cbm_ of 5%Cs. With the difference in work functions, the depletion zone from the contact between 10%Cs and 15%Cs created band bending that promotes electron moving from 15%Cs to 10%Cs. However, slightly lower potential E_cbm_ of 15%Cs compared to that of 10%Cs may induce electron-transporting into the unwanted direction. It is worth noting that, in Fig. 2g, the band diagrams of each layer of PPDs were arranged by equating  E_vac_ to zero, which may not represent the band diagram of PPDs when all layers are stacked.

From Fig. 2h, 5%Cs, 10%Cs, and 15%Cs have an emission peak at 783 nm, 769 nm, and 768 nm, respectively. The slight blueshift corresponds to a change in band gap. Double-layer perovskite 5%Cs/10%Cs has a PL peak located at 774 nm, which lays between those of 5%Cs and 10%Cs films, but the intensity was slightly lower. The quenching in PL intensity of the double-layer perovskite agrees with the band diagram in Fig. 2d; the heterojunction between 5%Cs and 10%Cs causes electrons to move from 10%Cs to 5%Cs better, thus increasing charge extraction rate and reducing radiative recombination^44,51^. This phenomenon was also true for 5%Cs/15%Cs and 10%Cs/15%Cs, which have emission peaks located at 770 nm and 768 nm, respectively. Both have lower emission intensities than those of their components alone. For 15%Cs/10%Cs, which was the inverse perovskite layer of 10%Cs and 15%Cs, the emission peak was located at 767 nm with the intensity higher than that of 10%Cs/15%Cs, indicating less charge extraction and higher recombination. Triple-layer 5%Cs/10%Cs/15%Cs had the emission peak located at 768 nm with intensity lower compared to those 5%Cs and 10%Cs, but slightly higher than that of 15%Cs. When swapped to 10%Cs and 15%Cs in the triple-layer perovskite to get 5%Cs/15%Cs/10%Cs, the intensity increased slightly as the result of less charge extraction and more recombination. The PL quenching of 5%Cs/15%Cs and 10%Cs/15%Cs was more significant than those of 5%Cs/10%Cs and 5%Cs/10%Cs/15%Cs which may relate to better charge extraction. Overall, varied PL magnitudes represented the interplay between charge extraction (3 layers > 2 layers > 1 layer), defect sites at the bulks (10%Cs ≈ 15%Cs > 5%Cs), and the interfacial defects (3 layers > 2 layers > 1 layer). The defect sites at the bulks (10%Cs ≈ 15%Cs > 5%Cs) are supported by PL-lifetime as shown in Fig. S7. With multiple layers, charge extraction was more effective at the cost of more interfacial defects being introduced. More defect sites cause higher rates of non-radiative recombination and depress PL intensities^10,52^.

External quantum efficiency or EQE(λ) was estimated using the equation:

$${\text{EQE}}\left( {\uplambda } \right) = \left( {\frac{{{\text{electrons}}/{\text{sec}}}}{{{\text{photons}}/{\text{sec}}}}} \right) \times 100 \%$$

**Figure 3:**
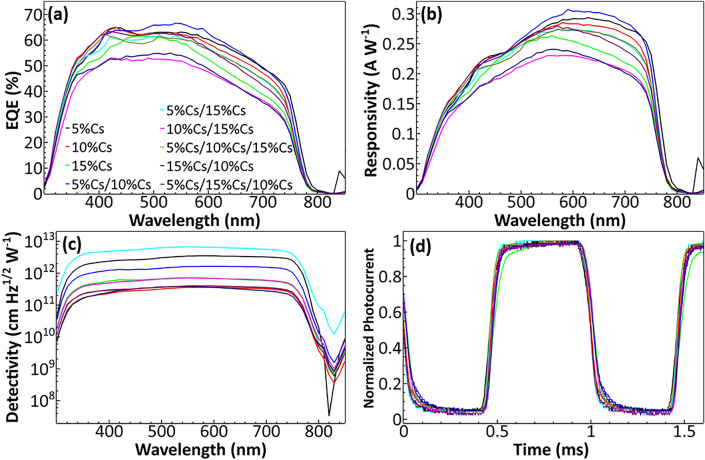
(**a**) EQE, (**b**) Responsivity, and (**c**) Detectivity spectra of single, double, and triple-layer perovskite PPDs. (**d**) Normalized photoresponse of PPDs under 1000 Hz optical chopper.

**Figure 4:**
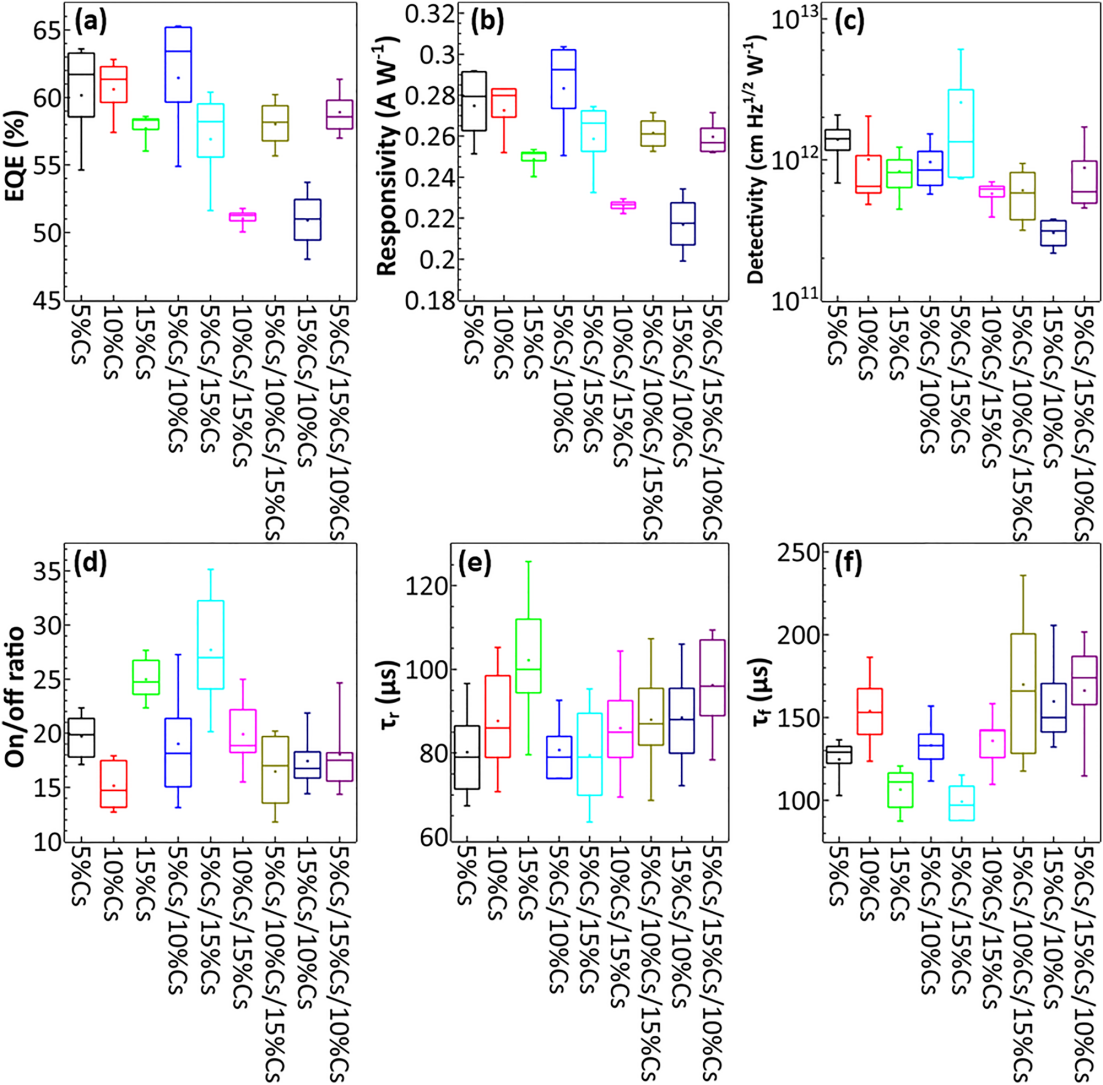
(**a**) EQE at 550 nm, (**b**) Responsivity at 600 nm, and (**c**) Specific detectivity at 550 nm of single, double, and triple-layer PPDs. (**d**) On/off ratio of single, double, and triple-layer PPDs. (**e**) Rising time (τ_r_) measured from 10 to 90% of maximum photocurrent of single, double, and triple-layer PPDs. (**f**) Falling time (τ_f_) measured from 90 to 10% of the maximum photocurrent of single, double, and triple-layer PPDs.

EQE determined the number of photoelectrons generated per number of photons. More photoelectrons generated translate to higher current. Since the number of electrons and the number of photons can be transformed into current and light intensity, respectively, and responsivity (R) is the product of those two parameters as shown in the equation:

$$R\left( \lambda \right) = \frac{I\left( \lambda \right)}{{P\left( \lambda \right)}}$$

where *I* is the photocurrent and *P* is light intensity; EQE and responsivity are directly correlated to each other. As shown in Fig. 3a,b, EQE and responsivity spectra of PPDs have similar trends to each other. PPDs with high EQE also have high responsivity. Assuming equal light intensity, PPDs with higher EQE and responsivity can produce higher photocurrent, which in solar cell application is very crucial in producing electricity. However, for photodetector application, the high current is not the main priority since in the real-world application only a small amount of current is needed for detection. The more crucial factor is specific detectivity (D^*^) as shown in Fig. 3c, which determined how well the photodetector can detect light at a lower intensity without being interfered with noise. The noise is the dark current that is generated even when there is no illumination and can be the result of current leakage in the device^53^. A higher noise level can overcome signals coming from low-intensity light, resulting in low D^*^. Specific detectivity can be determined by the equation:

$$D^{*} \left( \lambda \right) = \frac{R\left( \lambda \right)}{{\sqrt {2qJ_{d} } }}$$

where *J*_*d*_ is the dark current density. In general, we want EQE and responsivity (R) to be as high as possible while maximizing D^*^. Figure 3d provides normalized xenon lamp broad light photoresponse of PPDs under 1000 Hz optical chopper (Table 1). Another important factor for photodetectors is the detection range which can be determined from EQE or responsivity spectra. In broadband photodetector application, wider EQE and responsivity spectra are better. From Fig. 3a,b, the EQE and the responsivity of PPDs have high values covering from 350 to 750 nm, which fully cover the visible light spectrum making them suitable for broadband photodetector application. For EQE and responsivity, single, double, and triple-layer PPDs perform similarly as shown in Fig. 4a,b. Perovskite films from the spray process can have more surface defects than those from the spin process which is linked to the reduction in photocurrent^49,50^. We suspected that multilayer PPDs has better charge extraction, but high defects, resulting in less overall performance in term of photocurrent. All the measurements were done without external bias to emphasize the self-powering capability of the PPDs. From Table 2, J_sc_ of 5%Cs and 10%Cs are higher than those of multilayer PPDs except that of 5%Cs/10%Cs. Both 10%Cs/15%Cs and 15%Cs/10%Cs have significantly lower J_sc_, which can refer to non-ideal band alignment and too small energy differences (E_vbm_ and E_cbm_) between those two layers. Although no significant improvement in terms of photocurrent from multilayer PPDs was made, as seen in Table 1, our average responsivity is about 0.25, the record value for a self-powered detector fabricated by a scalable spray method. Nevertheless, this value is lower than R of 0.52 from the wasteful spin coating process^23^.

**Table 1:** Comparison of PPD parameters of different self-powered PPDs.

Device structure/fabrication process	R (A W^-1^)	EQE (%)	D* (cm Hz^1/2^ W^−1^)	On/off ratio	τ_r_/τ_f_	References
ITO/SnO_2_/5%Cs/15%Cs/Spiro/Au (spray coating for the absorber layer) (average value) (maximum values shown in Table S1)	0.2588	56.91	2.44 × 10^12^	28	80/99 μs	This work
FTO/c-TiO_2_/Cs_0.05_ MA_0.16_ FA_0.79_ Pb(I_0.9_Br_0.1_)_3_/Spiro/Au (spin coating)	0.52	~ 80	8.8 × 10^12^	7.3 × 10^5^	19/21 μs	^23^
ITO/CsPbBr_3_:ZnO/Ag (spin coating)	0.0115	–		12.86	409/17.92 ms	^35^
ITO/TiO_2_/MAPbI_3_/P3HT/Ag (spin coating)	0.16	–		4.89	1.2/0.2 s	^54^
ITO/SnO_2_/CsPbBr_3_(MC)/Spiro/Au (inverse temperature crystallization)	0.172	–	4.8 × 10^12^	1.3 × 10^5^	140/120 μs	^55^
FTO/CdS/MAPbI_3_/Spiro/Ag (spin coating)	0.48	–	2.1 × 10^13^	–	5.4/2.21 ms	^56^
FTO/C60/MAPbI_3_/GaN/In (dip coating)	0.198	–	7.96 × 10^12^	5 × 10^3^	45/63 ms	^57^
FTO/ MAPbBr_3_/MAPbI_x_Br_3−x_ /Au (inverse temperature crystallization)	0.0115	3.17		1.2	–/0.67 μs	^34^
ITO/SnO_2_/MAPbI_3_/Carbon (inverse temperature crystallization)	0.26	~ 52		2 × 10^5^	30/300 μs	^28^
FTO/TiO_2_/MAPbI_3_/Graphene/FTO (spin coating)	0.375	83	4.5 × 10^12^	4 × 10^6^	5 ms/5 ms	^58^
ITO/TiO_2_/PC_61_BM/MAPbI_3_/P3HT/MoO_3_/Ag (spin coating)	0.339	84	4.8 × 10^12^	~ 4 × 10^5^	–	^59^

**Table 2:** Average short circuit current density and dark current density of single, double, and triple-layer PPDs over 4 devices.

Samples	J_sc_ (mA cm^−2^)	J_d_ (nA cm^−2^)
5%Cs	13.39	1.283
10%Cs	13.13	1.011
15%Cs	12.05	1.611
5%Cs/10%Cs	13.30	0.639
5%Cs/15%Cs	12.29	0.259
10%Cs/15%Cs	10.97	1.572
5%Cs/10%Cs/15%Cs	12.64	1.079
15%Cs/10%Cs	10.47	2.033
5%Cs/15%Cs/10%Cs	12.50	2.351

From Fig. 4c, the D^*^ of 5%Cs/15%Cs has about one order of magnitude higher value compared to others as a result of its significantly lower dark current as shown in Table 3. The dark current can be lower by impeding charge carriers from moving in the opposite direction, typically done by adding barrier layers, which are electron blocking layer (hole transporting layer: HTL) and hole blocking layers (electron transporting layer: ETL)^54^. Alternatively, semiconductor heterojunction can be utilized for blocking holes from traveling in the reverse direction, replacing HTL^55^. However, surface defects also have an impact on the dark current of PPDs^56,57^. In our case, 5%Cs/15%Cs has a graded band structure that helps block holes from traveling from 15%Cs to 5%Cs, reducing the dark current. The work function difference between 5%Cs and 15%Cs is the largest among our heterojunction, creating the largest depletion zone and internal electric field pushing holes in the right direction. We suspected that the large depletion zone of 5%Cs/15%Cs can overcome the surface defect at the interlayer, resulting in low dark current and high detectivity. Moreover, among double-layer PPDs, 5%Cs/15%Cs has longest fast decay (τ_1_) which is associated with the lowest amount of trap-assisted recombination as shown in Fig. S7^9,60^. With the multi-layer materials 5%Cs/10%Cs and 5%Cs/15%Cs, τ_1_ becomes less and the fitting parameter B_1_ from Fig. S7 becomes larger close to those of 10%Cs and 15%Cs, implying more interface recombination from one more interface. Furthermore, 10%Cs/15%Cs has shorter τ_1_ compared to those of both 10%Cs and 15%Cs layers as an extra interface might enhance trap-assisted recombination.

**Table 3:** Averaged values of photodetector parameters for single, double, and triple-layer perovskite devices, over 8 devices for on/off ratios, τ_r_, and τ_f,_ and over 4 devices for EQE, R, and D*.

Samples	On/off ratio	τ_r_ (μs)	τ_f_ (μs)	EQE (550 nm) (%)	R (600 nm) (A W^-1^)	D* (550 nm) (cmHz^1/2^ W^−1^)
5%Cs	20	80	125	60.18	0.2748	1.39 × 10^12^
10%Cs	15	88	154	60.62	0.2726	1.01 × 10^12^
15%Cs	25	103	107	57.73	0.2488	8.28 × 10^11^
5%Cs/10%Cs	19	87	147	61.46	0.2834	9.65 × 10^11^
5%Cs/15%Cs	28	80	99	56.91	0.2588	2.44 × 10^12^
10%Cs/15%Cs	20	86	136	51.06	0.2261	5.77 × 10^11^
5%Cs/10%Cs/15%Cs	16	88	170	58.04	0.2617	6.09 × 10^11^
15%Cs/10%Cs	17	89	160	50.92	0.2170	3.04 × 10^11^
5%Cs/15%Cs/10%Cs	18	96	166	58.92	0.2597	8.82 × 10^11^

Specific detectivity also correlated to the on/off ratio of PPDs from measuring the photoresponse dynamics. In Fig. 4d, on/off ratios of 5%Cs/10%Cs and 10%Cs/15%Cs were similar to that of 5%Cs, which showed no significant improvement as the effect of additionally electric field strength might not be able to overcome the drawbacks from more interfacial defect sites created from the additional interface. Yet the improvement of double-layer PPDs could be observed in 5%Cs/15%Cs as the on/off ratio was noticeably higher than those of 5%Cs and 15%Cs; the self-powered champion device was able to achieve the on/off ratio of 35.5. Moreover, 5%Cs/15%Cs also has the lowest τ_r_ and τ_f_ as shown in Fig. 4e, f with the champion achieving 70 μs for the rising time and 88 μs for the falling time. In comparison, the single-layer 5%Cs PPD can achieve 70 μs for the rising time and 98μ for the falling time. The internal electric field generated from the depletion zone also promotes the velocity of carriers, resulting in faster response speed^30^. For other multilayer PPDs, the internal electric field is not adequate. Surface defects at the interlayer cause response speed to decrease especially falling time^49^. The increase in rising and falling times is more prominent in triple-layer PPDs as these devices have one more interlayer than double-layer PPDs, thus more area containing surface defects. Only 5%Cs/15%Cs PPD has the internal electric field strong enough to significantly improve response speed over the drawback from surface defects.

The stability of the perovskite films and the PPDs are shown in Fig. S8 and S9. We observe no Cs redistribution as there are no new peaks from CsI or Cs-containing phases in the XRD patterns, possibly due to large activation energy of A-site cation to from vacancy and/or interstitial defects unlike the anion site^60,61^.

## Conclusions

The band diagram of triple cation perovskite could be altered by changing the Cs^+^ ratio within the perovskite crystal. With the increase of Cs^+^, the work function increased; thus when connected, multilayer perovskite induced band bending, which facilitated electron transportation through drift transport that was significantly faster when compared to diffusion transport. Faster electron transportation, therefore, results in faster response speeds (τ_r_ and τ_f_). However, multilayer perovskite films have more surface defect sites which may hurt the performance of PPDs. The double-layer 5%Cs/15%Cs PPD struck the right balance between maximizing performance from grading structure and introducing interfacial defect sites. The 5%Cs/15%Cs PPD provided faster speed and better detectivity as the result of lower dark current compared to those of single-layer PPDs in agreement with the correct band alignment. More interfacial defects in triple-layer PPDs caused small or no improvement from those of single-layer PPDs.

## Supplementary Information

Supplementary Information.

## Data Availability

The data that support the findings of this study are available from the corresponding author upon reasonable request.

## References

[1] Shaikh JS, Shaikh NS, Sheikh AD, Mali SS, Kale AJ, Kanjanaboos P, Hong CK, Kim JH, Patil PS (2017). Perovskite solar cells: in pursuit of efficiency and stability. Mater. Des..

[2] Veldhuis SA, Boix PP, Yantara N, Li M, Sum TC, Mathews N, Mhaisalkar SG (2016). Perovskite materials for light-emitting diodes and lasers. Adv. Mater..

[3] Jung HS, Park NG (2015). Perovskite solar cells: from materials to devices. Small.

[4] Boix PP, Agarwala S, Koh TM, Mathews N, Mhaisalkar SG (2015). Perovskite solar cells: beyond methylammonium lead iodide. J. Phys. Chem. Lett..

[5] Afzaal M, Yates HM (2017). Growth patterns and properties of aerosol-assisted chemical vapor deposition of CH_3_NH_3_PbI_3_ films in a single step. Surf. Coat. Technol..

[6] Pramchu S, Jaroenjittichai AP, Laosiritaworn Y (2016). Surface doping of Sn in orthorhombic CH_3_NH_3_PbI_3_ for potential perovskite solar cells: first principles study. Surf. Coat. Technol..

[7] Boonthum C, Pinsuwan K, Ponchai J, Srikhirin T, Kanjanaboos P (2018). Reconditioning perovskite films in vapor environments through repeated cation doping. Appl. Phys. Express..

[8] Pinsuwan K, Boonthum C, Supasai T, Sahasithiwat S, Kumnorkaew P, Kanjanaboos P (2019). Solar perovskite thin films with enhanced mechanical, thermal, UV, and moisture stability via vacuum-assisted deposition. J. Mater. Sci..

[9] Ponchai J, Kaewurai P, Boonthum C, Pinsuwan K, Supasai T, Sahasithiwat S, Kanjanaboos P (2019). Modifying morphology and defects of low-dimensional, semi-transparent perovskite thin films: via solvent type. RSC Adv..

[10] Kaewurai P, Ponchai J, Amratisha K, Naikaew A, Swe KZ, Pinsuwan K, Boonthum C, Sahasithiwat S, Kanjanaboos P (2019). Enhancing violet photoluminescence of 2D perovskite thin films via swift cation doping and grain size reduction. Appl. Phys. Express..

[11] Ponchai J, Srathongsian L, Amratisha K, Boonthum C, Sahasithiwat S, Ruankham P, Kanjanaboos P (2021). Modified colored semi-transparent perovskite solar cells with enhanced stability. J. Alloys Compds..

[12] Bishop JE, Read CD, Smith JA, Routledge TJ, Lidzey DG (2020). Fully spray-coated triple-cation perovskite solar cells. Sci. Rep..

[13] Chen H, Ding X, Pan X, Hayat T, Alsaedi A, Ding Y, Dai S (2018). Comprehensive studies of air-brush spray deposition used in fabricating high-efficiency CH_3_NH_3_PbI_3_ perovskite solar cells: combining theories with practices. J. Power Sources.

[14] Liang Z, Zhang S, Xu X, Wang N, Wang J, Wang X, Bi Z, Xu G, Yuan N, Ding J (2015). A large grain size perovskite thin film with a dense structure for planar heterojunction solar cells via spray deposition under ambient conditions. RSC Adv..

[15] Adnan M, Irshad Z, Lee JK (2020). Facile all-dip-coating deposition of highly efficient (CH_3_)_3_NPbI_3-x_Cl_x_ perovskite materials from aqueous non-halide lead precursor. RSC Adv..

[16] Tong S, Gong C, Zhang C, Liu G, Zhang D, Zhou C, Sun J, Xiao S, He J, Gao Y, Yang J (2019). Fully-printed, flexible cesium-doped triple cation perovskite photodetector. Appl. Mater. Today.

[17] Sutherland BR, Johnston AK, Ip AH, Xu J, Adinolfi V, Kanjanaboos P, Sargent EH (2015). Sensitive, fast, and stable perovskite photodetectors exploiting interface engineering. ACS Photonics.

[18] Bao C, Chen Z, Fang Y, Wei H, Deng Y, Xiao X, Li L, Huang J (2017). Low-noise and large-linear-dynamic-range photodetectors based on hybrid-perovskite thin-single-crystals. Adv. Mater..

[19] Xu X, Chueh CC, Jing P, Yang Z, Shi X, Zhao T, Lin LY, Jen AKY (2017). High-performance near-IR photodetector using low-bandgap MA_0.5_FA_0.5_Pb_0.5_Sn_0.5_I_3_ perovskite. Adv. Funct. Mater..

[20] Cheng CC, Zhan JY, Liao YM, Lin TY, Hsieh YP, Chen YF (2016). Self-powered and broadband photodetectors based on graphene/ZnO/silicon triple junctions. Appl. Phys. Lett..

[21] Ou Q, Bao X, Zhang Y, Shao H, Xing G, Li X, Shao L, Bao Q (2019). Band structure engineering in metal halide perovskite nanostructures for optoelectronic applications. Nano Mater. Sci..

[22] Yang J, Yu T, Zhu K, Xu Q (2017). High-gain and fast-response metal-semiconductor-metal structured organolead halide perovskite photodetectors. J. Phys. D: Appl. Phys..

[23] Adams GR, Eze VO, Shohag MAS, Simpson R, Parker H, Okoli OI (2020). Fabrication of rapid response self-powered photodetector using solution-processed triple cation lead-halide perovskite. Eng. Res. Express.

[24] Zalewski EF, Duda CR (1983). Silicon photodiode device with 100% external quantum efficiency. Appl. Opt..

[25] Miao J, Zhang F (2019). Recent progress on highly sensitive perovskite photodetectors. J. Mater. Chem. C..

[26] Periyanagounder D, Gnanasekar P, Varadhan P, He JH, Kulandaivel J (2018). High performance, self-powered photodetectors based on a graphene/silicon Schottky junction diode. J. Mater. Chem. C.

[27] Lim JW, Wang H, Choi CH, Kwon H, Quan LN, Park WT, Noh YY, Kim DH (2019). Self-powered reduced-dimensionality perovskite photodiodes with controlled crystalline phase and improved stability. Nano Energy.

[28] Pan X, Zhou H, Liu R, Wu D, Song Z, Tang X, Yang X, Wang H (2020). Achieving a high-performance, self-powered, broadband perovskite photodetector employing MAPbI3 microcrystal films. J. Mater. Chem. C.

[29] di Bartolomeo A (2016). Graphene Schottky diodes: an experimental review of the rectifying graphene/semiconductor heterojunction. Phys. Rep..

[30] Ou Q, Zhang Y, Wang Z, Yuwono JA, Wang R, Dai Z, Li W, Zheng C, Xu ZQ, Qi X, Duhm S, Medhekar NV, Zhang H, Bao Q (2018). Strong depletion in hybrid perovskite p–n junctions induced by local electronic doping. Advanced Materials..

[31] Lee CH, Lee GH, van der Zande AM, Chen W, Li Y, Han M, Cui X, Arefe G, Nuckolls C, Heinz TF, Guo J, Hone J, Kim P (2014). Atomically thin p–n junctions with van der Waals heterointerfaces. Nat. Nanotechnol..

[32] Li B, Zhang Y, Zhang L, Yin L (2017). Graded heterojunction engineering for hole-conductor-free perovskite solar cells with high hole extraction efficiency and conductivity. Adv. Mater..

[33] Wang H, Wang X, Chen Y, Zhang S, Jiang W, Zhang X, Qin J, Wang J, Li X, Pan Y, Liu F, Shi Z, Zhang H, Tu L, Wang H, Long H, Li D, Lin T, Wang J, Zhan Y, Shen H, Meng X, Chu J (2020). Extremely low dark current MoS2 photodetector via 2D halide perovskite as the electron reservoir. Adv. Opt. Mater..

[34] Cao M, Tian J, Cai Z, Peng L, Yang L, Wei D (2016). Perovskite heterojunction based on CH_3_NH_3_PbBr_3_ single crystal for high-sensitive self-powered photodetector. Appl. Phys. Lett..

[35] Li C, Han C, Zhang Y, Zang Z, Wang M, Tang X, Du J (2017). Enhanced photoresponse of self-powered perovskite photodetector based on ZnO nanoparticles decorated CsPbBr_3_ films. Sol. Energy Mater. Sol. Cells.

[36] Mehmood U, Al-Ahmed A, Afzaal M, Al-Sulaiman FA, Daud M (2017). Recent progress and remaining challenges in organometallic halides based perovskite solar cells. Renew. Sustain. Energy Rev..

[37] Hong QM, Xu RP, Jin TY, Tang JX, Li YQ (2019). Unraveling the light-induced degradation mechanism of CH_3_NH_3_PbI_3_ perovskite films. Organ. Electron.: Phys. Mater. Appl..

[38] Ouafi M, Jaber B, Atourki L, Bekkari R, Laânab L (2018). Improving UV stability of MAPbI3 perovskite thin films by bromide incorporation. J. Alloy. Compd..

[39] Saliba M, Matsui T, Seo JY, Domanski K, Correa-Baena JP, Nazeeruddin MK, Zakeeruddin SM, Tress W, Abate A, Hagfeldt A, Grätzel M (2016). Cesium-containing triple cation perovskite solar cells: Improved stability, reproducibility and high efficiency. Energy Environ. Sci..

[40] Wang Y, Wu J, Zhang P, Liu D, Zhang T, Ji L, Gu X, David Chen Z, Li S (2017). Stitching triple cation perovskite by a mixed anti-solvent process for high performance perovskite solar cells. Nano Energy..

[41] Jiang Y, Leyden MR, Qiu L, Wang S, Ono LK, Wu Z, Juarez-Perez EJ, Qi Y (2018). Combination of hybrid CVD and cation exchange for upscaling Cs-substituted mixed cation perovskite solar cells with high efficiency and stability. Adv. Funct. Mater..

[42] Amratisha K, Ponchai J, Kaewurai P, Pansa-ngat P, Pinsuwan K, Kumnorkaew P, Ruankham P, Kanjanaboos P (2020). Layer-by-layer spray coating of a stacked perovskite absorber for perovskite solar cells with better performance and stability under a humid environment. Opt. Mater. Express..

[43] Pansa-Ngat P, Nakajima H, Supruangnet R, Suwanna S, Pakawatpanurut P, Sahasithiwat S, Kanjanaboos P (2021). Phase evolution in lead-free Cs-doped FASnI_3 _Hybrid perovskites and optical properties. J. Phys. Chem. C.

[44] Sun H, Deng K, Xiong J, Li L (2020). Graded bandgap perovskite with intrinsic n–p homojunction expands photon harvesting range and enables all transport layer-free perovskite solar cells. Adv. Energy Mater..

[45] Zhang T, Wu J, Zhang P, Ahmad W, Wang Y, Alqahtani M, Chen H, Gao C, Chen ZD, Wang Z, Li S (2018). High speed and stable solution-processed triple cation perovskite photodetectors. Adv. Opt. Mater..

[46] Thirumoorthi M, Thomas Joseph Prakash J (2016). Structure, optical and electrical properties of indium tin oxide ultra thin films prepared by jet nebulizer spray pyrolysis technique. J. Asian Ceram. Soc..

[47] Ašmontas S, Čerškus A, Gradauskas J, Grigucevičienė A, Leinartas K, Lučun A, Petrauskas K, Selskis A, Sužiedėlis A, Širmulis E, Juškėnas R (2021). Cesium-containing triple cation perovskite solar cells. Coatings.

[48] Eperon GE, Stranks SD, Menelaou C, Johnston MB, Herz LM, Snaith HJ (2014). Formamidinium lead trihalide: a broadly tunable perovskite for efficient planar heterojunction solar cells. Energy Environ. Sci..

[49] Xu Z, Teo SH, Gao L, Guo Z, Kamata Y, Hayase S, Ma T (2019). La-doped SnO_2_ as ETL for efficient planar-structure hybrid perovskite solar cells. Org. Electron..

[50] Zhao YQ, Liu B, Yu ZL, Cao D, Cai MQ (2017). Tuning charge carrier types, superior mobility and absorption in lead-free perovskite CH_3_NH_3_GeI_3_: theoretical study. Electrochim. Acta.

[51] Cao F, Wang M, Li L (2020). Graded energy band engineering for efficient perovskite solar cells. Nano Select..

[52] Swe KZ, Naikaew A, Kaewurai P, Pansa-Ngat P, Sahasithiwat S, Kangkaew L, Rugmai S, Soontaranon S, Kanjanaboos P (2020). Layered perovskite with compact morphology and reduced grain size via vacuum assisted crystallization for luminescence applications. Opt. Mater. Express..

[53] Simone G, Dyson MJ, Meskers SCJ, Janssen RAJ, Gelinck GH (2020). Organic photodetectors and their application in large area and flexible image sensors: the role of dark current. Adv. Funct. Mater..

[54] Lu H, Tian W, Cao F, Ma Y, Gu B, Li L (2016). A self-powered and stable all-perovskite photodetector-solar cell nanosystem. Adv. Func. Mater..

[55] Zhou H, Zeng J, Song Z, Grice CR, Chen C, Song Z, Zhao D, Wang H, Yan Y (2018). Self-powered all-inorganic perovskite microcrystal photodetectors with high detectivity. J. Phys. Chem. Lett..

[56] Cao F, Meng L, Wang M, Tian W, Li L (2019). Gradient energy band driven high-performance self-powered perovskite/CdS photodetector. Adv. Mater..

[57] Zhou H, Mei J, Xue M, Song Z, Wang H (2017). High-stability, self-powered perovskite photodetector based on a CH_3_NH_3_PbI_3_/GaN heterojunction with C60 as an electron transport layer. J. Phys. Chem. C.

[58] Li J, Yuan S, Tang G, Li G, Liu D, Li J, Hu X, Liu Y, Li J, Yang Z, Liu SF, Liu Z, Gao F, Yan F (2017). High-performance, self-powered photodetectors based on perovskite and graphene. ACS Appl. Mater. Interfaces..

[59] Liu C, Wang K, Yi C, Shi X, Du P, Smith AW, Karim A, Gong X (2015). Ultrasensitive solution-processed perovskite hybrid photodetectors. J. Mater. Chem. C..

[60] Jin H, Debroye E, Keshavarz M, Scheblykin IG, Roeffaers MBJ, Hofkens J, Steele JA (2020). It’s a trap! on the nature of localised states and charge trapping in lead halide perovskites. Mater. Horiz..

[61] Ghasemi M, Zhang L, Yun JH, Hao M, He D, Chen P, Bai Y, Lin T, Xiao M, Du A, Lyu M, Wang L (2020). Dual-ion-diffusion induced degradation in lead-free Cs_2_AgBiBr_6_ double perovskite solar cells. Adv. Funct. Mater..

