# Supplementary material for: Graded multilayer triple cation perovskites for high speed and detectivity self-powered photodetector via scalable spray coating process
Source: Sci Rep. 2022 Jun 30;12:11058. doi: 10.1038/s41598-022-14774-x (PMC9247054; doi:10.1038/s41598-022-14774-x)
Supplement: Supplementary file 1 — Supplementary Information. [file 41598_2022_14774_MOESM1_ESM.docx]

**Graded Multilayer Triple Cation Perovskites for High Speed and Detectivity Self-Powered Photodetector via Scalable Spray Coating Process**

Koth Amratishaa, Waris Tuchindaa, Pipat Ruankhamb,c, Atittaya Naikaewa, Pimsuda Pansa-Ngata, Ladda Srathongsiana, Worawat Wattanathana^d^, Ko Ko Shin Thanta, Ratchadaporn Supruangnete, Hideki Nakajimae, Somboon Sahasithiwatf, Pongsakorn Kanjanaboosa,g,*∗*

*aSchool of Materials Science and Innovation, Faculty of Science, Mahidol University, Nakhon Pathom, 73170, Thailand*

*bDepartment of Physics and Materials Science, Faculty of Science, Chiang Mai University, Chiang Mai, 50200, Thailand*

*^c^Research Center in Physics and Astronomy, Faculty of Science, Chiang Mai University, Chiang Mai, 50200, Thailand*

*dDepartment of Materials Engineering, Faculty of Engineering, Kasetsart University, Bangkok, 10900, Thailand*

*eSynchrotron Light Research Institute (Public Organization), Nakhon Ratchasima, 30000, Thailand*

*fNational Metal and Materials Technology Center (MTEC), National Science and Technology Development Agency, Pathum Thani, 12120, Thailand*

*gCenter of Excellence for Innovation in Chemistry (PERCH-CIC), Ministry of Higher Education, Science, Research and Innovation, Bangkok, 10400, Thailand*

*∗Corresponding author email address:* [Pongsakorn.kan@mahidol.edu](mailto:Pongsakorn.kan@mahidol.edu)

**Supplemental Materials**


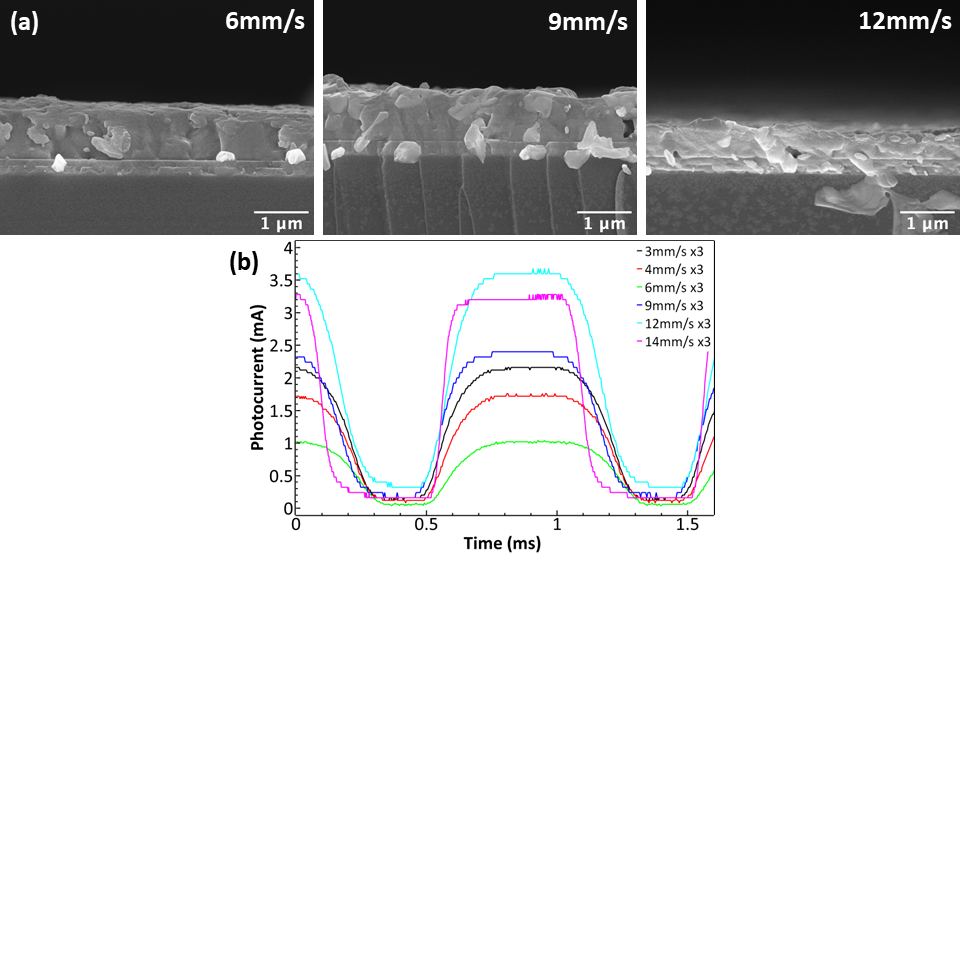


**Figure S1.** (a) SEM cross-section of triple-layer 5%Cs/10%Cs/15%Cs perovskite films fabricated with the airbrush speeds of 6mm/s, 9mm/s, and 12mm/s. (b) Photoresponse of triple-layer 5%Cs/10%Cs/15%Cs PPDs fabricated with different airbrush speeds.

By changing the airbrush speeds, the thickness of the resulting film can be changed. Slower airbrush speeds deposit more solution onto the substrate, resulting in a thicker film as shown in Figure S1 (a). In terms of performance, we observed two trends for the relationship between airbrush speed and photocurrent of PPDs. At slow speed from 3 mm/s to 6mm/s the performance of the PPDs increases with thickness. However, at higher speeds from 9mm/s to 12mm/s, the performance decreases with thickness. As the thicknesses typically change along with the morphological changes, a simple correlation cannot be made. The optimal airbrush speed was deduced to be 12mm/s.


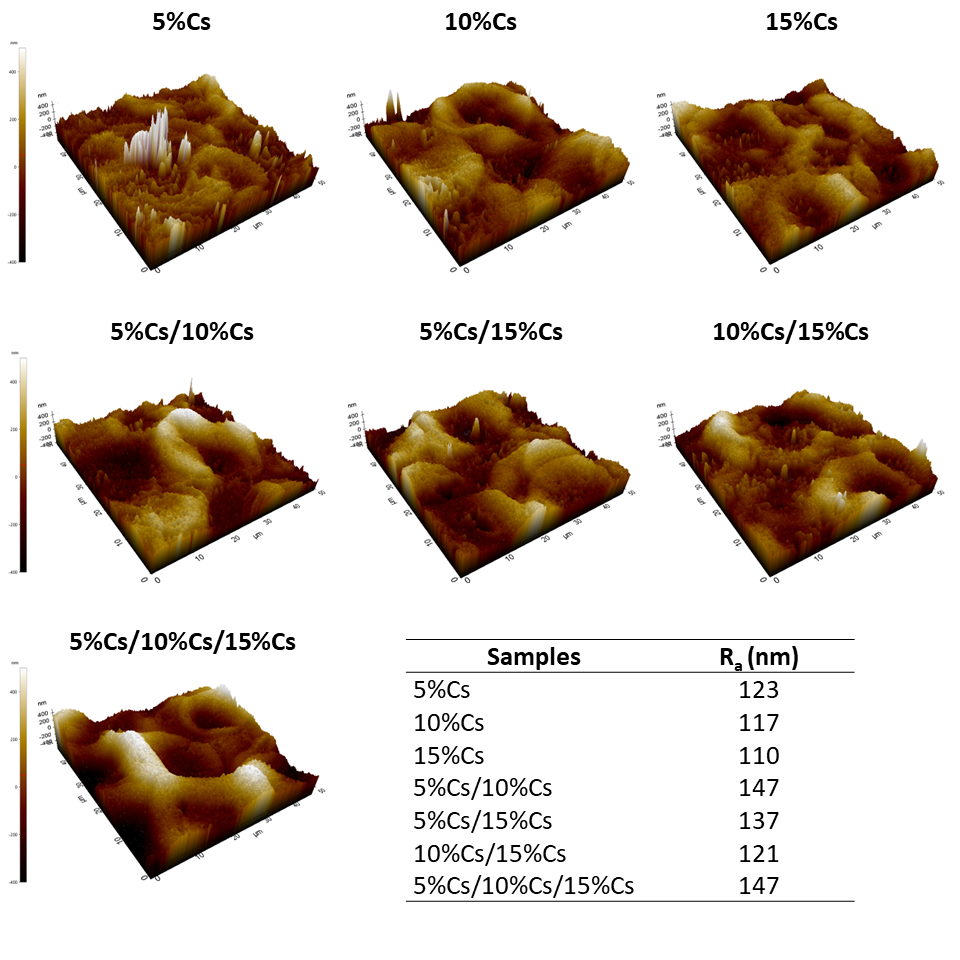


**Figure S2.** Surface topology of single, double, and triple-layer perovskite films. The

table below shows the root mean square of the z height of the perovskite film.

From AFM results, we can see the coffee ring morphological structure. This structure was most likely created when the solution droplets during spray deposition beads up due to surface tension. These characteristics increased the overall roughness of the perovskite films. Most likely, the coffee ring caused the reduction in performance of PPDs and the effect is slightly stronger for multi-layer PPDs. The disadvantage of rougher surface finish from the presented spray deposition compared to conventional methods such as spin coating can be compensated by the spray coating’s ability to produce multilayer perovskite films, easily, cheaply, and inexpensively. According to the Figure S2, the root means square (R_a_) of perovskite films is less than 150 nm which is less than the thickness of perovskite film (>600 nm, see Figure 1 in the main text). Therefore, an assumption can be made that the manufactured perovskite films have almost zero empty areas where the ETL layer is exposed.

**
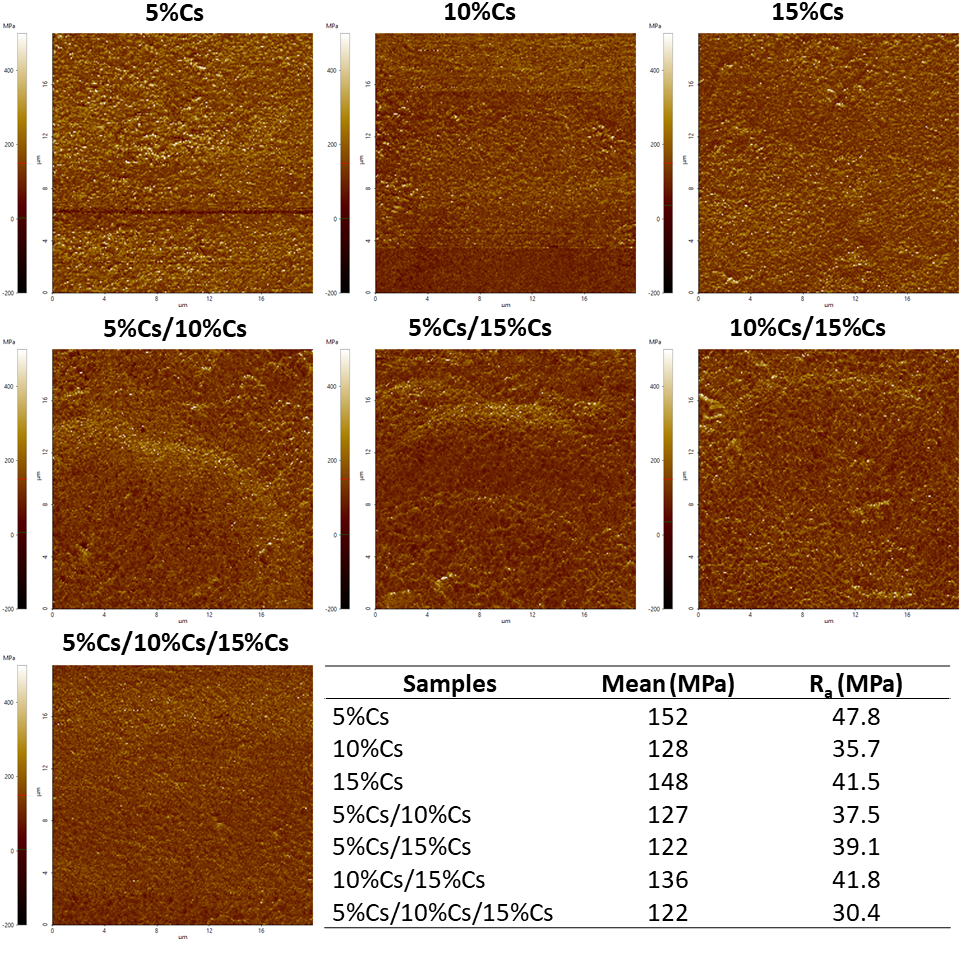
**

**Figure S3.** Surface modulus mapping of single, double, and triple-layer perovskite films. The table below shows the mean and the root mean square of the modulus values.

We studied the homogeneity of the perovskite films using modulus mapping from AFM. From the Figure S5, we observed homogenous modulus values across the surface of the spray coated perovskite films. Note that the modulus values from AFM are good for the comparative study only as the absolute values can be hugely different due to a lot of assumptions used in the modulus estimation. However, the experiments were done back-to-back for all conditions, using the same AFM cantilever and the same AFM settings.


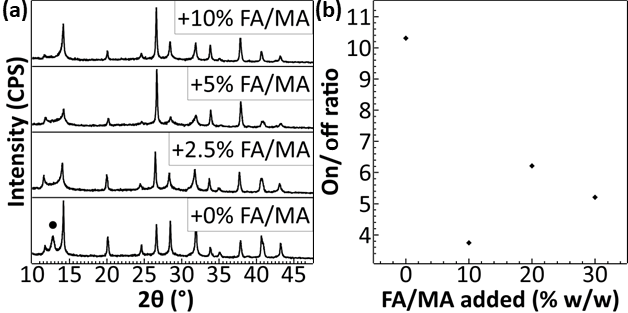


**Figure S4.** (a) X-ray diffraction of triple cation perovskite films deposited using precursor solutions with extra FA/MA cation to reduce PbI_2_ content. PbI_2_ peak is marked with •. (b) On/off ratio of triple cation perovskite films deposited using precursor solutions with extra FA/MA cation.


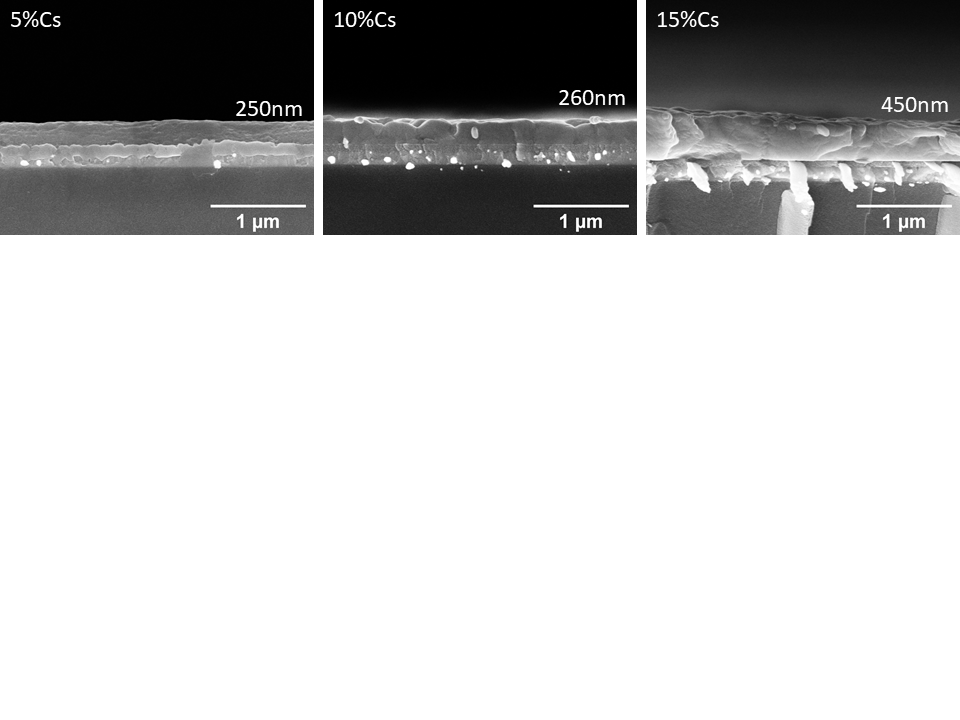


**Figure S5.** Cross-sections of 5%Cs, 10%Cs, and 15%Cs triple cation perovskite films fabricated with the speed of 9mm/s for 1 time.


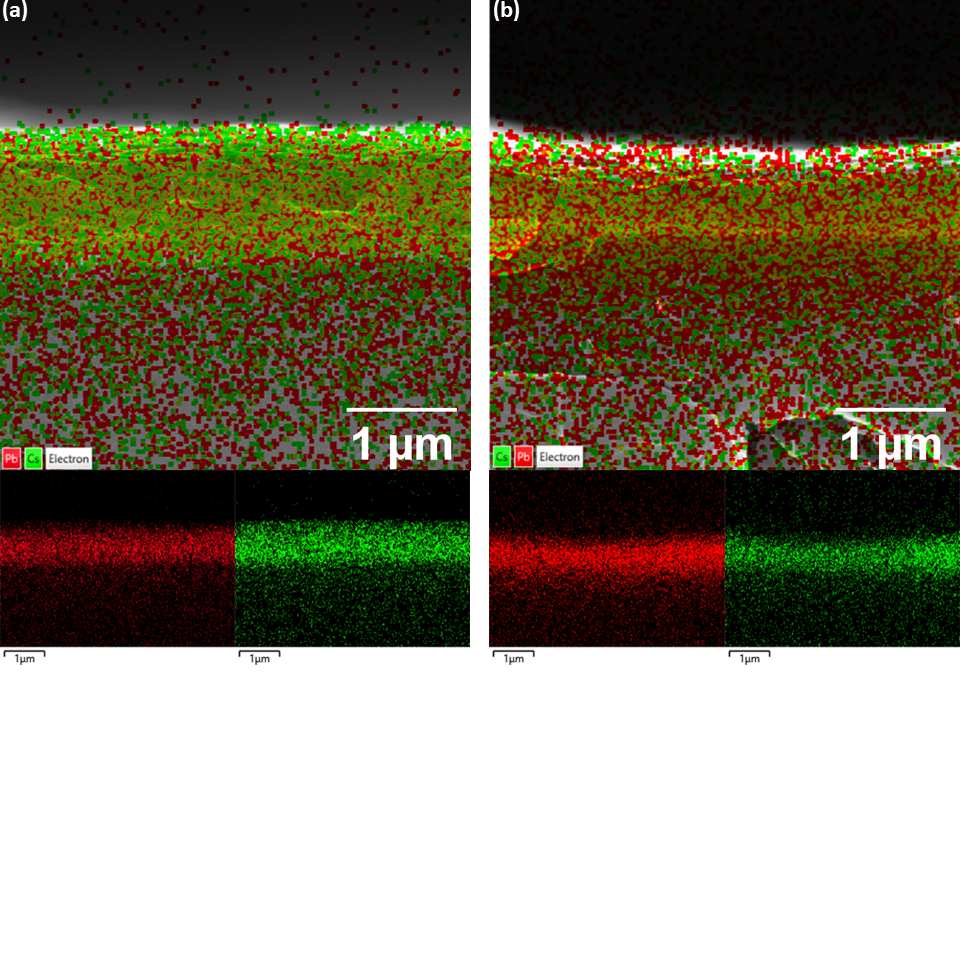


**Figure S6.** Elemental analysis at the SEM cross-section of (a) double-layer 5%Cs/15%Cs and (b) triple-layer 5%Cs/15%Cs/10%Cs perovskite films. Cs is green and Pb is red.

**
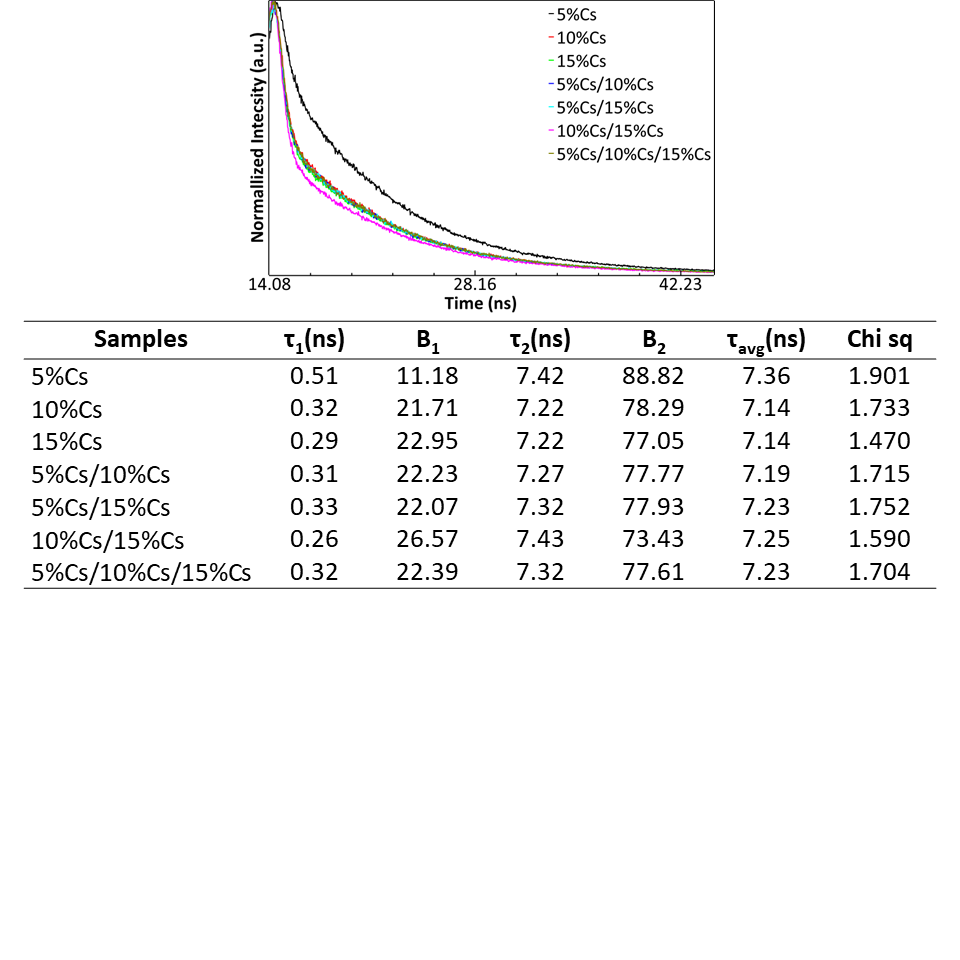
**

**Figure S7.** PL-lifetime results of single, double, and triple-layer perovskite films. The data can be fit to determine the lifetimes (τ_1_, τ_2_) and pre-exponential values (B_1_, B_2_).

For single-layer perovskite film, 5%Cs has the longest τ_1_ which is associated with low amount of trap-assisted recombination [1,2] followed by 10%Cs and 15%Cs. We assumed that the low concentration of trap-assisted recombination of 5%Cs causes double-layer 5%Cs/10%Cs and 5%Cs/15%Cs to have relatively long τ_1_ as both samples contain 5%Cs layer. The double-layer 10%Cs/15%Cs has the shortest τ_1_, because it contains 10%Cs, and 15%Cs layers which have shorter τ_1_. Furthermore, 10%Cs/15%Cs has shorter τ_1_ compared to those of both 10%Cs and 15%Cs layers as an extra interface might enhance trap-assisted recombination. With the multi-layer materials 5%Cs/10%Cs and 5%Cs/15%Cs, τ_1_ becomes less and B_1_ becomes larger close to those of 10%Cs and 15%Cs, implying more recombination from one more interface.

The lifetime τ_1_, τ_2_ were obtained by fitting the actual data using the equation[3]:

$$I=\sum_{i}^{n} \alpha_{i}\exp\left( -\frac{t}{\tau_{i}} \right)$$

Where $\alpha_{1}=B_{1}/(B_{1}+B_{2})$, and $\alpha_{2}=B_{2}/(B_{1}+B_{2})$. The average lifetimes (τ_avg_) were obtained using the equation[3]:

$$\tau_{avg}={\sum_{i=1}^{n} \alpha_{i}\tau_{i}^{2}}/{\sum_{i=1}^{n} \alpha_{i}\tau_{i}}$$

**
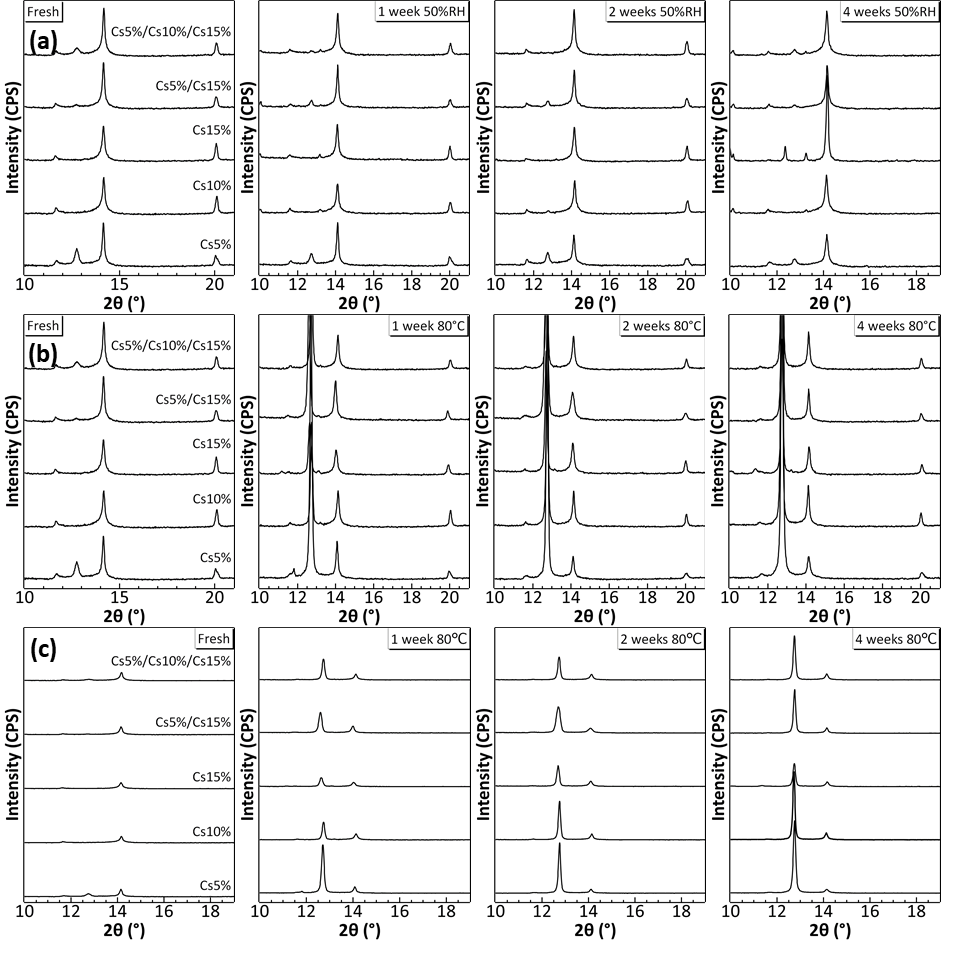
**

**Figure S8.** (a) XRD results of degraded perovskite films under 50%RH humidity. (b) XRD results of degraded perovskite films under 80°C inside an inert, N_2_ glovebox environment. (c) Narrow view XRD results of degraded perovskite films under 80°C inside the N_2_ glovebox environment, for observing the evolution of PbI_2_ peaks at 12.7°.

We observed similar XRD patterns in fresh, 1-, 2-, and 4-week films, stored in 50%RH ambient environment. The results showed that the α-phase at 14.2° still clearly presented over the course of 4-week stability test. It indicated that the fabricated films had high resistance to moisture. On the other hand, the perovskite films could not tolerate heat over time because the PbI_2_ peak at 12.7° significantly emerged as shown in Figure S8 (b,c). According to the stability results, the fabricated films can greatly withstand moisture, however, tend to degrade to PbI_2_ with exposure to 80°C over a month.

**
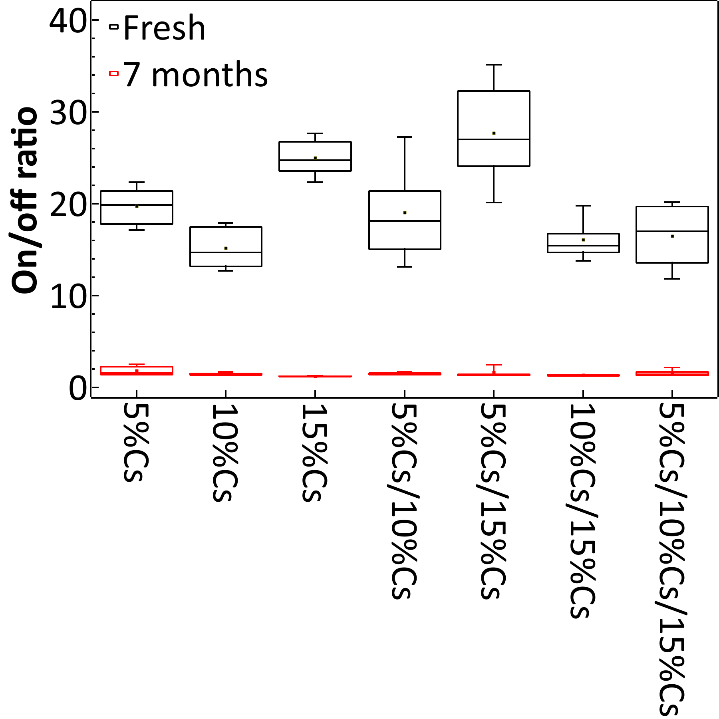
**

**Figure S9.** On/off ratio of single, double, and triple-layer PPDs right after fabrication, compared to 7 months after fabrication inside a low humidity (50%RH) environment.

From Figure S9, the on/off ratio of PPDs after 7 months is significantly lower than those of the fresh devices. The degradation of PPDs were expected as the devices were exposed to humidity without encapsulation. Multi-layer PPDs do not demonstrate higher stability in agreement with the film stability results shown in Figure S8.

**Table S1.** Raw data of EQE(550nm), R(600nm), D(550nm), J_sc_, and J_d_ obtained from EQE measurements.

| Samples | EQE (%) | R (A/W) | D* (cmHz^1/2^W^-1^) | J_sc_(mA cm^-2^) | J_d_(nA cm^-2^) |
| --- | --- | --- | --- | --- | --- |
| 5%Cs | 53.64 | 0.248 | 1.45E+12 | 12.19 | 1.898 |
|  | 63.15 | 0.292 | 5.63E+11 | 14.29 | 3.131 |
|  | 63.67 | 0.291 | 2.19E+12 | 13.89 | 0.087 |
|  | 60.25 | 0.268 | 1.38E+12 | 13.20 | 0.018 |
| 10%Cs | 62.08 | 0.283 | 6.28E+11 | 13.67 | 0.596 |
|  | 56.85 | 0.248 | 4.58E+11 | 12.13 | 0.064 |
|  | 60.61 | 0.277 | 6.67E+11 | 13.41 | 0.000 |
|  | 62.94 | 0.283 | 2.28E+12 | 13.55 | 3.385 |
| 15%Cs | 58.63 | 0.254 | 3.97E+11 | 12.33 | 2.825 |
|  | 58.32 | 0.252 | 9.06E+11 | 12.18 | 0.000 |
|  | 55.64 | 0.238 | 1.29E+12 | 11.64 | 2.500 |
|  | 58.33 | 0.252 | 7.22E+11 | 12.03 | 1.120 |
| 5%Cs/10%Cs | 65.30 | 0.304 | 9.94E+11 | 14.26 | 1.540 |
|  | 65.17 | 0.301 | 1.62E+12 | 14.05 | 0.059 |
|  | 61.68 | 0.283 | 5.48E+11 | 13.34 | 0.567 |
|  | 53.71 | 0.245 | 6.99E+11 | 11.54 | 0.391 |
| 5%Cs/15%Cs | 60.61 | 0.275 | 7.65E+11 | 13.18 | 0.254 |
|  | 59.16 | 0.272 | 2.69E+11 | 13.03 | 0.390 |
|  | 57.25 | 0.261 | 6.82E+12 | 12.11 | 0.004 |
|  | 50.64 | 0.227 | 1.92E+12 | 10.85 | 0.390 |
| 10%Cs/15%Cs | 51.86 | 0.230 | 7.11E+11 | 11.10 | 1.019 |
|  | 51.30 | 0.227 | 6.29E+11 | 11.07 | 0.832 |
|  | 51.24 | 0.226 | 3.55E+11 | 10.97 | 2.284 |
|  | 49.84 | 0.222 | 6.13E+11 | 10.74 | 2.152 |
| 5%Cs/10%Cs/15%Cs | 57.30 | 0.252 | 3.01E+11 | 12.28 | 1.794 |
|  | 60.41 | 0.273 | 4.02E+11 | 13.16 | 1.128 |
|  | 59.05 | 0.266 | 9.73E+11 | 12.85 | 0.799 |
|  | 55.38 | 0.257 | 7.60E+11 | 12.28 | 0.595 |
| 15%Cs/10%Cs | 47.67 | 0.197 | 2.10E+11 | 9.59 | 3.869 |
|  | 50.08 | 0.211 | 3.82E+11 | 10.15 | 0.909 |
|  | 51.92 | 0.225 | 2.59E+11 | 10.84 | 2.180 |
|  | 54.03 | 0.236 | 3.66E+11 | 11.31 | 1.174 |
| 5%Cs/15%Cs/10%Cs | 61.74 | 0.273 | 1.89E+12 | 13.19 | 2.347 |
|  | 59.13 | 0.261 | 4.46E+11 | 12.65 | 3.944 |
|  | 58.01 | 0.253 | 5.11E+11 | 12.19 | 1.918 |
|  | 56.81 | 0.252 | 6.77E+11 | 11.98 | 1.195 |

**Table S2.** Raw data of on/off ratio, rising time, and falling time obtained from photoresponse measurements.

| Samples | On/Off ratio | τ_r_ (μs) | τ_f_ (μs) |
| --- | --- | --- | --- |
| 5%Cs | 17.00 | 74 | 126 |
|  | 22.67 | 72 | 112 |
|  | 21.25 | 84 | 98 |
|  | 18.50 | 94 | 132 |
|  | 21.75 | 66 | 130 |
|  | 21.25 | 70 | 138 |
|  | 18.00 | 84 | 134 |
|  | 17.33 | 98 | 128 |
| 10%Cs | 12.80 | 76 | 194 |
|  | 12.67 | 82 | 172 |
|  | 13.33 | 100 | 166 |
|  | 14.60 | 108 | 160 |
|  | 17.75 | 68 | 118 |
|  | 14.83 | 80 | 146 |
|  | 18.00 | 90 | 134 |
|  | 17.40 | 98 | 142 |
| 15%Cs | 22.50 | 130 | 98 |
|  | 22.25 | 102 | 110 |
|  | 27.00 | 110 | 118 |
|  | 25.00 | 118 | 122 |
|  | 26.67 | 74 | 86 |
|  | 24.50 | 90 | 90 |
|  | 24.00 | 96 | 112 |
|  | 22.50 | 98 | 116 |
| 5%Cs/10%Cs | 15.33 | 78 | 184 |
|  | 14.33 | 90 | 180 |
|  | 12.50 | 94 | 180 |
|  | 16.67 | 102 | 140 |
|  | 26.50 | 74 | 116 |
|  | 19.67 | 80 | 128 |
|  | 27.67 | 84 | 116 |
|  | 19.60 | 96 | 134 |
| 5%Cs/15%Cs | 35.50 | 70 | 88 |
|  | 26.67 | 74 | 88 |
|  | 31.50 | 88 | 102 |
|  | 34.50 | 96 | 110 |
|  | 23.50 | 60 | 88 |
|  | 24.33 | 70 | 92 |
|  | 18.33 | 84 | 118 |
|  | 27.33 | 94 | 108 |
| 10%Cs/15%Cs | 19.00 | 80 | 142 |
|  | 16.80 | 88 | 144 |
|  | 18.75 | 92 | 142 |
|  | 14.80 | 110 | 166 |
|  | 18.75 | 66 | 104 |
|  | 25.00 | 76 | 120 |
|  | 25.00 | 82 | 128 |
|  | 21.25 | 94 | 142 |
| 5%Cs/10%Cs/15%Cs | 11.17 | 86 | 232 |
|  | 19.60 | 86 | 124 |
|  | 15.00 | 88 | 182 |
|  | 13.80 | 108 | 190 |
|  | 20.30 | 70 | 114 |
|  | 20.00 | 68 | 130 |
|  | 19.00 | 92 | 150 |
|  | 13.00 | 106 | 238 |
| 15%Cs/10%Cs | 17.50 | 84 | 142 |
|  | 20.67 | 80 | 128 |
|  | 16.00 | 92 | 146 |
|  | 17.33 | 106 | 154 |
|  | 22.50 | 68 | 140 |
|  | 16.20 | 80 | 164 |
|  | 15.60 | 92 | 190 |
|  | 13.80 | 106 | 214 |
| 5%Cs/15%Cs/10%Cs | 18.00 | 96 | 174 |
|  | 15.25 | 106 | 204 |
|  | 17.50 | 108 | 158 |
|  | 27.33 | 76 | 96 |
|  | 18.40 | 84 | 158 |
|  | 16.00 | 94 | 178 |
|  | 14.00 | 110 | 196 |

**References**

[1] J. Ponchai, P. Kaewurai, C. Boonthum, K. Pinsuwan, T. Supasai, S. Sahasithiwat, P. Kanjanaboos, Modifying morphology and defects of low-dimensional, semi-transparent perovskite thin films via solvent type, RSC Advances. 9 (2019) 12047–12054. https://doi.org/10.1039/c9ra00971j.

[2] X. He, K.A. Velizhanin, G. Bullard, Y. Bai, J.H. Olivier, N.F. Hartmann, B.J. Gifford, S. Kilina, S. Tretiak, H. Htoon, M.J. Therien, S.K. Doorn, Solvent- and wavelength-dependent photoluminescence relaxation dynamics of carbon nanotube sp^3^ defect states, ACS Nano. 12 (2018) 8060–8070. https://doi.org/10.1021/acsnano.8b02909.

[3] A practical guide to time-resolved luminescence lifetime determination using dedicated time-correlated single-photon counting systems by HORIBA Jobin Yvon IBH Ltd.
